# Amino acids at the exposed C-terminus of the S coat protein of cowpea mosaic virus play different roles in particle formation and viral systemic movement

**DOI:** 10.1099/jgv.0.001285

**Published:** 2019-06-06

**Authors:** Yulia Meshcheriakova, George P. Lomonossoff

**Affiliations:** 1 Department of Biological Chemistry, John Innes Centre, Norwich Research Park, Colney Ln, Norwich, NR4 7UH, UK

**Keywords:** Cowpea mosaic virus, virus-like particles, systemic movement, C-terminus S protein

## Abstract

The icosahedral capsid of cowpea mosaic virus is formed by 60 copies of the large (L) and small (S) coat protein subunits. The 24-amino-acid C-terminal peptide of the S coat protein can undergo proteolytic cleavage without affecting particle stability or infectivity. Mutagenic studies have shown that this sequence is involved in particle assembly, virus movement, RNA encapsidation and suppression of gene silencing. However, it is unclear how these processes are related, and which part(s) of the sequence are involved in each process. Here, we have analysed the effect of mutations in the C-terminal region of the S protein on the assembly of empty virus-like particles and on the systemic movement of infectious virus. The results confirmed the importance of positively charged amino acids adjacent to the cleavage site for particle assembly and revealed that the C-terminal 11 amino acids are important for efficient systemic movement of the virus.

Cowpea mosaic virus (CPMV), the type member of the comovirus genus of plant viruses, has a single-stranded, positive-sense, RNA genome. The two genomic RNA molecules (RNA-1 and RNA-2) are separately encapsidated within icosahedral particles, consisting of 60 copies each of the large (L) and small (S) coat proteins, which are produced by proteolytic processing of a precursor, VP60 [1, 2]; the structure of the virions is known to atomic resolution [3]. Virus preparations can be resolved into two electrophoretic forms, fast (f) and slow (s) [4], depending on the presence or absence of 24 amino acids at the carboxyl-terminus of the S subunits; cleavage leaves L189 as the C-terminal amino acid in the majority of the f form particles [5–78]. The region that is cleaved consists of two distinct domains: a portion proximal to the site of cleavage between L190 and R202 that contains six basic amino acids (4R and 2K) and a distal region of 11 amino acids between S203 and A213 (the C-terminal amino acid of the S protein) that is essentially uncharged, containing only a single histidine (Table 1).

**Table 1.** Deletion and substitution mutants in the 24-amino-acid cleavable C-terminus of S coat protein of CPMV The site of cleavage after L189 is indicated by / and the arginines (R) that are specifically mutated are shown in **bold**. The effect of the mutations on the assembly of empty virus-like particles (eVLPs) after transient expression and on the ability of infectious virus to move systemically is shown. WT indicates the properties of the mutants are similar to that of WT virus and – indicates the effect on systemic movement has not been determined. *The ability of mutant ∆11 to form eVLPs has been reported previously by Hesketh *et al*. [8].

**Mutant**	**Assembly of eVLPs**	**Systemic movement**
**WT** 193195 199 202 L/LKF**R**F**R**DIE**R**SK**R**SVMVGHTATAA	WT	WT
**∆11** L/LKF**R**F**R**DIE**R**SK**R**	WT*	NO
**R193/195** **G** L/LKF**G**F**G**DIE**R**SK**R**SVMVGHTATAA	No	–
**R199E/R202D** L/LKF**R**F**R**DIE**E**SK**D**SVMVGHTATAA	Expanded	WT
**R195G** L/LKF**R**F**G**DIE**R**SK**R**SVMVGHTATAA	WT	–
**R195/199/202** **G** L/LKF**R**F**G**DIE**G**SK**G**SVMVGHTATAA	Very poor	–
**R193G** L/LKF**G**F**R**DIE**R**SK**R**SVMVGHTATAA	No	–
**Δ24–3** **R** L/**RRR**	WT	NO
**Δ24–5** **R** L/**RRRRR**	WT	–
**R199/202** **G** L/LKF**R**F**R**DIE**G**SK**G**SVMVGHTATAA	WT	–
**∆R193** L/LKF∆F**R**DIE**R**SK**R**SVMVGHTATAA	Very poor	–
**∆ K201/R202** L/LKF**R**F**R**DIE**R**S**∆∆**SVMVGHTATAA	WT	–
**Δ24-3R+** **11** L/**RRR**SVMVGHTATAA	WT	WT

The loss of carboxyl-terminal amino acids from the S subunits does not affect the integrity of particles as the f and s form particles have identical specific infectivity [9, 10] and RNA isolated from both forms was shown to be intact [10]. This suggested that the 24-amino-acid sequence plays no role in maintaining the structure and integrity of the mature virus particle, a suggestion supported by x-ray crystallographic studies on the virus in which the 24 amino acids were not visible [3]. Taylor *et al*. [6] produced a series of mutants in which amino acids were sequentially deleted from the C-terminus of the S protein and examined the yield and ability of the virus to move systemically following infection of cowpea plants (*Vigna unguiculata*). Deletions within the 24-amino-acid segment resulted in mutants with reduced yield and ability to spread both locally and systemically, with the degree of debilitation depending on the size of the deletion. The mutant in which the entire 24 amino acids were deleted (DM4), though viable, appeared to be defective in RNA encapsidation [6]; deletions beyond this point resulted in mutants that were not viable. Subsequent studies indicated that the C-terminal region also plays a role in the suppression of gene silencing [11].

Though demonstrating that the C-terminal region plays an important part in the viral replication cycle, the studies with infectious virus could not distinguish whether this was a result of a debilitation of virus assembly, efficiency of virus spread or a combination of the two. The development of a system for the production of CPMV empty virus-like particles (eVLPs) through transient expression of coat protein precursor, VP60, and the viral 24K protease necessary for its processing [2] has enabled the process of capsid formation to be studied in the absence of infection. Such studies showed that while deletion of the entire 24-amino-acid segment severely disrupted eVLP formation [8, 12], deletion of 11 amino acids from the C-terminus (mutant Δ11; Table 1) had no effect [8, 13], indicating that the determinants of efficient capsid formation lie entirely between L190 and R202, the region containing the six basic amino acids. Cryo-electron microscopy of eVLPs revealed that this sequence lies between adjacent S subunits around the fivefold icosahedral axis of assembled particles and that R193 can form a salt bridge with E147 of the neighbouring S subunit [8]. Disruption of this salt bridge by mutagenesis prevents capsid formation, a process that can be reversed by the introduction of a compensatory mutation. However, efficient assembly is not entirely governed by the presence or absence of R193 since deletions of greater than 14 amino acids, also debilitated assembly [8]. An alternative explanation that mutations in the C-terminus of the S protein somehow affect processing at the L-S junction by the 24K protease seems unlikely since such processing can occur efficiently when the 24K recognition site is placed at different positions within the RNA-2-encoded polyprotein [14]. Furthermore, an entire protein can be placed at the end of the S protein via a 2A catalytic peptide without affecting particle formation [15], a rather more drastic change than the mutations introduced in the current studies.

To define the roles of the basic amino acids in the region between L190 and R202, mutations were introduced into this region of pEAQ-*HT*-VP60 [2] using the GENEART Site-Directed Mutagenesis System (Invitrogen). The resulting constructs were electroporated into *Agrobacterium tumefaciens* strain LBA4404 and the bacterial suspensions co-infiltrated with pEAQ-*HT*-24K into leaves of 3-week-old *Nicotiana benthamiana* plants as previously described [16]. *N. benthamiana* leaves were harvested 5–7 days post-infiltration and the ability of the mutant proteins to assemble into eVLPs was assessed by examining particle preparations extracted from the infiltrated leaves by SDS-PAGE and transmission electron microscopy (TEM) of samples negatively stained with 2 % (w/v) uranyl acetate [8, 13]. As previously shown above (Fig. 1a), mutant ∆11 assembles to give eVLPs as efficiently as the WT coat protein as judged by the levels of the cleaved L and S subunits in the stained SDS-PAGE gel and accumulation of typical CPMV particles (Fig. 1a). In the mutants where R193 was either mutated to G or deleted (R193G and ∆R193), no or very reduced levels of particles could be purified (Table 1; Fig. 1b), consistent with the previous identification of this amino acid as critical for eVLP assembly [8]. Simultaneous mutation of the three downstream arginines to glycine (R195/199/202G) also adversely affected assembly showing that the presence of R193 alone is necessary but not sufficient to allow assembly (Fig. 1b). By contrast, mutation of just two of these arginines to either glycine (R199/R202G) or acidic amino acids (R199E/R202D) resulted in protein that was assembly-competent, as did mutation of R195 alone to G in R195G (Table 1; Fig. 1b). Simultaneous deletion of K201 and R202 (∆K201/R202) also permitted particle assembly (Table 1). The particles from R199E/R202D appeared to have a greater diameter (37–45 nm; Fig. 1b) than those of WT eVLPs or the other mutants (30 nm; for an enlarged image showing the diameters of individual particles, see Fig. S1, available in the online version of this article). This effect is most probably caused by differential interaction with the negative stain during the transmission electron microscopy (TEM) analysis as a result of the negative charges on the particle surface, since cryo-EM analysis showed that the overall structure of the particles was unchanged (E.L. Hesketh, personal communication). The results confirm the importance of R193 but indicate that additional positive charges near L189 are also necessary for efficient eVLP formation. To confirm this hypothesis, the entire 24-amino-acid sequence was replaced with a sequence of either three or five arginines to give mutants Δ24–3R and Δ24–5R, respectively (Table 1). Both mutants were found to assemble efficiently to give eVLPs, with the yield obtained with Δ24–3R appearing to be slightly higher than that obtained with Δ24–5R and very similar to that found with WT eVLPs (Table 1; Fig. 1c) as judged by SDS-PAGE analysis of the L and S proteins in particle preparations.

**Fig. 1.**
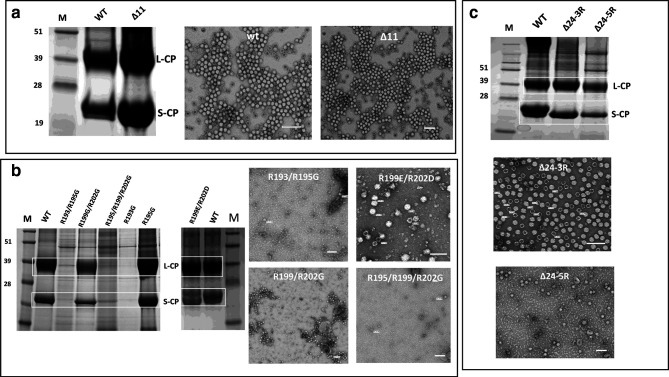
Ability of mutants to form eVLPs. The ability of VP60 from each mutant to be correctly processed to the L and S proteins and form eVLPs was assessed by SDS-PAGE of denatured particles and electron microscopy of virus preparations from infiltrated leaves. (a) Comparison of the ability of WT and mutant Δ11 to form eVLPs. (b) Assembly properties of selected arginine mutants. The level of cleaved L and S protein in the mutants compared to those found in WT eVLPs produced in the same experiment is shown in the SDS-PAGE gels. (c) Assembly properties of mutants Δ24–3R and Δ24–5R, with the levels of the L and S proteins also compared to that of WT eVLPs. For the SDS-PAGE gels, the positions of the L (L-CP) and S (S-CP) coat proteins are indicated on the right-hand side. In (b) and (c) the positions of L-CP and S-CP across the tracks is additionally highlighted by white boxes. The markers are shown in the lanes marked M with sizes shown on the left-hand side of the gel. Scale bars in electron micrographs represent 100 nm.

The finding that neither deletion of the C-terminal 11 amino acids nor the replacement of the entire 24-amino-acid segment by three or five arginine residues negatively impacts particle assembly, raises the question as to the function(s) of other parts of the C-terminal sequence, particularly the region from S203 to A213, which is not visible in cryo-EM reconstructions of eVLPs [8]. To investigate whether this region plays any role in the viral replication cycle, the coat protein sequences from Δ−11, Δ24–3R or R199E/R202D, all of which can assemble efficiently to form eVLPs, were substituted for the WT coat proteins in the infectious cDNA clone of CPMV RNA-2, pEAQ-RNA-2 [8]. The modified or WT pEAQ-RNA-2 sequences were agro-infiltrated into groups of five *N. benthamiana* in the presence of pBinPS1NT, which contains a full-length copy of RNA-1 [17]. Virus preparations made from the infiltrated leaves showed the presence of the mature L and S subunits and assembled virus particles by SDS-PAGE and TEM analysis, respectively, (Fig. 2a) indicating the ability of the mutants to assemble was preserved during infection. Analysis of the RNA encapsidated within the particles showed they all had WT levels of both RNA-1 and RNA-2, demonstrating they are competent for RNA encapsidation (data not shown). However, only WT and R199E/R202D were able to cause symptoms and the accumulation of viral particles in the systemic leaves of infiltrated plants by 14 days post-infiltration (days p.i.) (Fig. 2a). As in the case of eVLP formation, the particles produced from R199E/R202D had a larger diameter than those from WT virus (Fig. 2b).

**Fig. 2.**
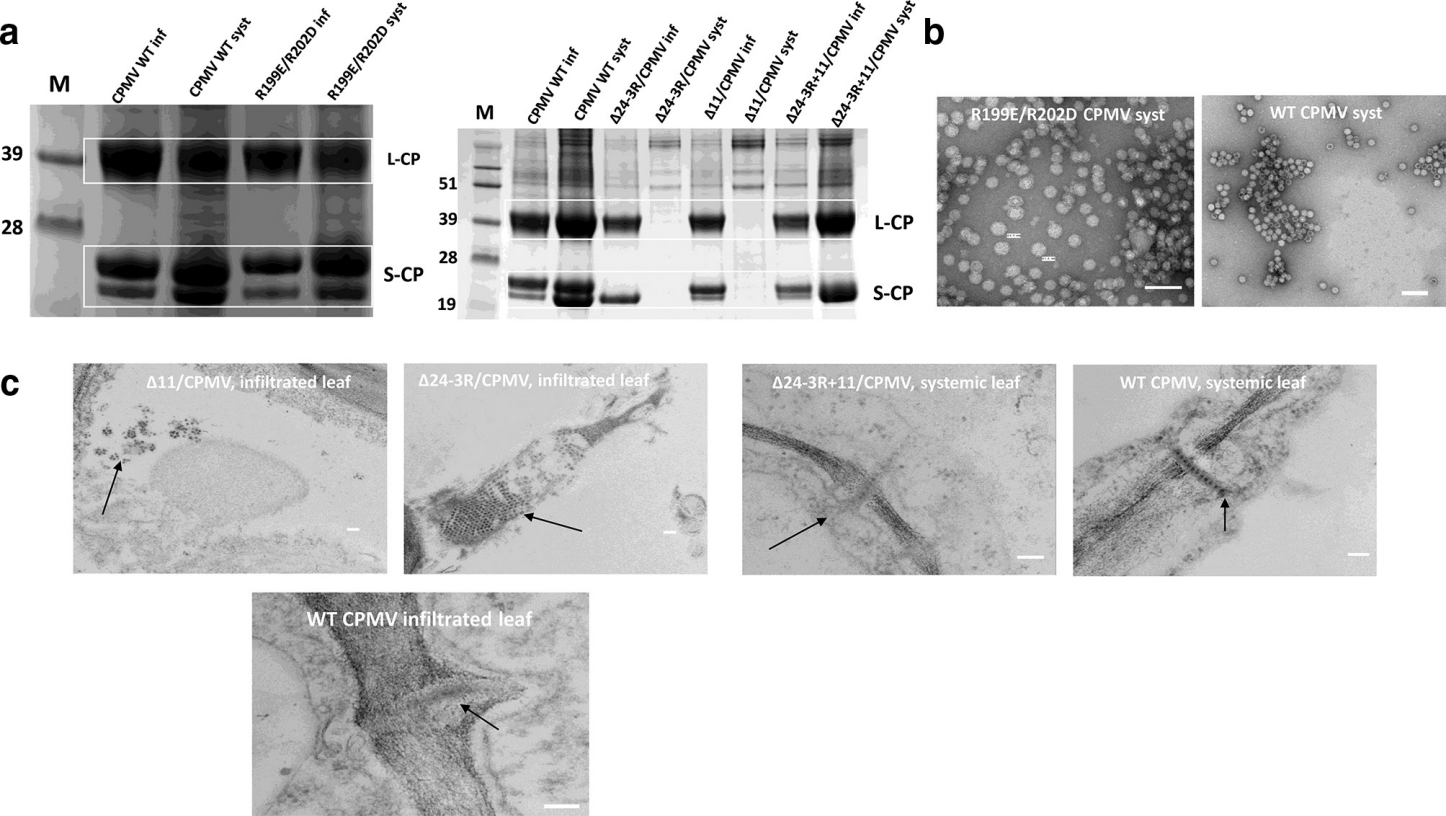
Ability of mutant virus particles to infect plants and move systemically. (a) SDS-PAGE analysis of virus preparations from infiltrated (inf) or systemic leaves (syst) of *N. benthamiana*. The positions of the L (L-CP) and S (S-CP) coat proteins are indicated on the right-hand side and highlighted by white boxes. The markers are shown in the lanes marked M with sizes shown on the left-hand side of the gel. (b) Electron micrograph of particles isolated from the systemic leaves of plants infiltrated with R199E/R202D. (c) Thin sections of infiltrated and systemic leaves of plants infiltrated with the designated mutants compared with that of WT virus. Scale bars in electron micrographs represent 100 nm.

The above results suggest that the exposure of positive charges on the surface of particles may have a deleterious effect on their ability to move systemically, an effect counteracted by changing two of the basic amino acids to acidic residues in the case of R199E/R202D. To investigate whether the natural C-terminal amino acids could serve this function, a further mutant, Δ24-3R+11, was created in which the sequence encoding S203 to A213 was placed immediately downstream of the three arginines in Δ24–3R in pEAQ-RNA-2. Plants inoculated with either wt RNA-2 or Δ24-3R+11 developed systemic symptoms by 14 days p.i. and particles could be extracted from the symptomatic leaves (Fig. 2a). These observations, coupled with the transient expression studies on eVLP assembly, suggest that while the presence of basic amino acids adjacent to L189 is sufficient to allow efficient particle assembly, exposure of these positively charged amino acids on the particle surface interferes with the ability of the virus to spread. This effect can be relieved by including the sequence from S203 to A213 downstream of the basic sequence.

To address the mechanism by which amino acids 203–213 can alleviate the systemic movement-deficient phenotype of Δ−11 and Δ24–3R, samples of leaves from plants infiltrated with WT RNA-2, Δ−11, Δ24–3R or Δ24-3R+11 in the presence of RNA-1 were fixed in a solution of 2.5 % (v/v) glutaraldehyde in 0.05M sodium cacodylate, pH 7.3, post-fixed with 1 % (w/v) osmium tetroxide and embedded in LR white resin. After polymerization, the resulting material was sectioned with a diamond knife using a Leica EM UC6 ultramicrotome (Leica Microsystems), the sections contrast-stained with 2 % (w/v) uranyl acetate and 1 % (w/v) lead citrate for 1 h and analysed by TEM. Examination of the samples from leaves infiltrated with Δ−11 or Δ24–3R revealed the presence of aggregates of virus particles. Such aggregates were not seen in the equivalent samples from leaves infiltrated with WT RNA-2 or Δ24-3R+11. (Fig. 2c); by contrast, particles could be seen in plasmodesmata in samples from both the infiltrated and systemically infected upper leaves of plants, a situation consistent with the ability of these constructs to efficiently move systemically (Fig. 2c). The appearance of aggregates of virus particles, coupled with an impaired ability to move systemically, in the case of Δ−11 and Δ24–3R is reminiscent of the situation found with chimaeric CPMV particles bearing positively charged insertions within the surface-exposed βB-βC or βC′-βC" loop of the S protein [18]. In the case of the chimaeras displaying positively charged peptides, the defect in virus movement could be relieved by incorporating acidic amino acids in the same or an adjacent loop [19] and it appears that the sequence from S203 to A213 at the C-terminus of the S protein serves in a similar function in WT virus.

The data presented here support the notion that there are two distinct domains within the C-terminal region of CPMV – a basic region proximal to the cleavage site (amino acids 190–202) involved in particle formation and a distal region (amino acids 203–213) involved in efficient movement of the virus. The role of basic amino acids in particle formation is consistent with the conclusions of previous cryo-EM and mutagenesis studies [8, 13]. The current studies show that the precise order of basic amino acids is not critical, and that the native sequence can be replaced by three or five arginines without abrogating eVLP formation. Indeed, the substitution of the natural sequence with three arginines allows the formation of RNA-containing particles suggesting that assembly proceeds entirely normally in this case. On the other hand, deletion of amino acids 203–213, though having no effect on particle formation, results in particles which aggregate within infected cells and which cannot spread systemically. These amino acids are not visible in the cryo-EM reconstruction of eVLPs, suggesting that they may be unstructured or adopt a variety of conformations. Movement of CPMV from cell-to-cell and systemically requires the presence of the both RNA-2-encoded 48K movement protein as well as the coat proteins [20], with assembled particles moving through plasmodesmata modified by the 48K protein [21]. It is tempting to conclude that the formation of aggregates within infected cells sequesters particles rendering them unavailable for interaction with plasmodesmata. However, Porta *et al*. [18] reported that chimaeras that were movement-competent also formed aggregates so the relationship between aggregation and lack of movement is unclear. An alternative explanation is that excess positive charge on the particle surface inhibits a productive interaction with the 48K protein within the plasmodesmata. The region of the 48K protein that interacts with virions has been mapped to the C-terminal 48 amino acids of the protein [22–24], though this appeared to interact with the L rather than the S protein [23]. However, these findings do not exclude the possibility that additional exposed positive charges derived from the S protein could interfere with the binding of assembled virus particles. It is also possible that alterations within the C-terminal region could unmask some feature on the particle surface that triggers a plant response, limiting virus movement. Indeed, a necrotic response was noted in cowpeas infected with mutant DM4 [6], though the restriction of systemic movement was not as absolute as observed in the current studies.

## Supplementary Data

Supplementary material 1
