# Supplementary material for: Amino acids at the exposed C-terminus of the S coat protein of cowpea mosaic virus play different roles in particle formation and viral systemic movement
Source: J Gen Virol. 2019 Jun 6;100(7):1165–70. doi: 10.1099/jgv.0.001285 (PMC7414441; doi:10.1099/jgv.0.001285)
Supplement: Supplementary material 1 [file jgv-100-1165-s001.pdf]

Δ24-3R/eVLPs

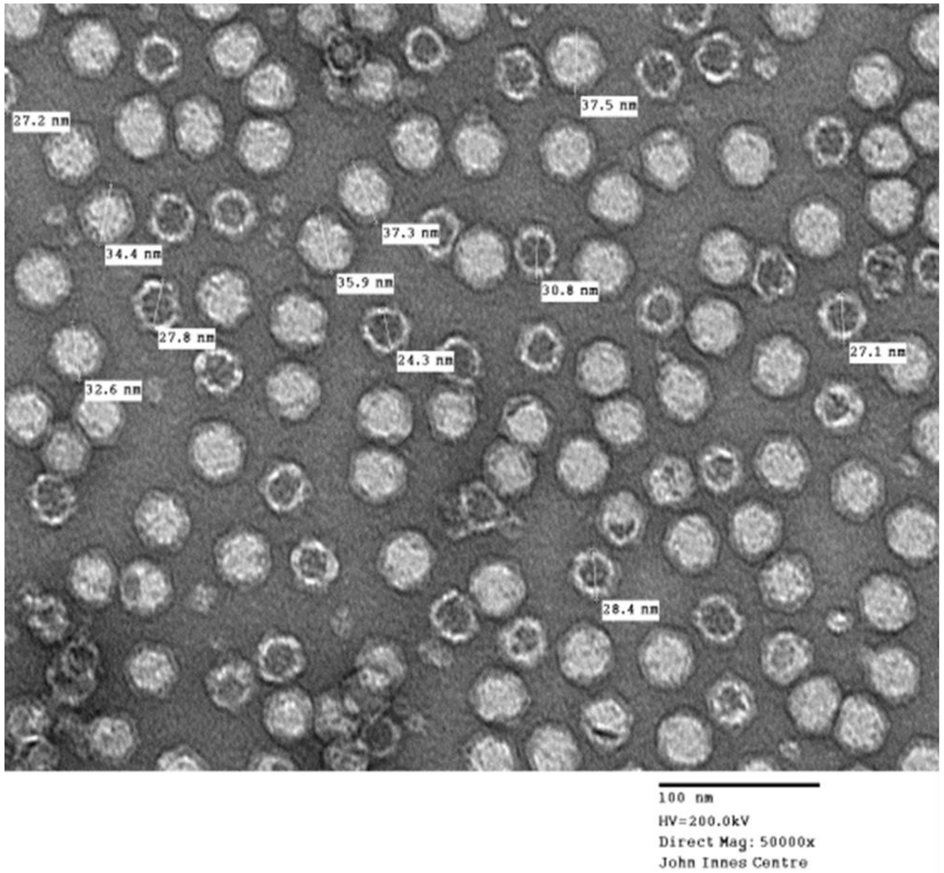

R199E/R202D/eVLPs

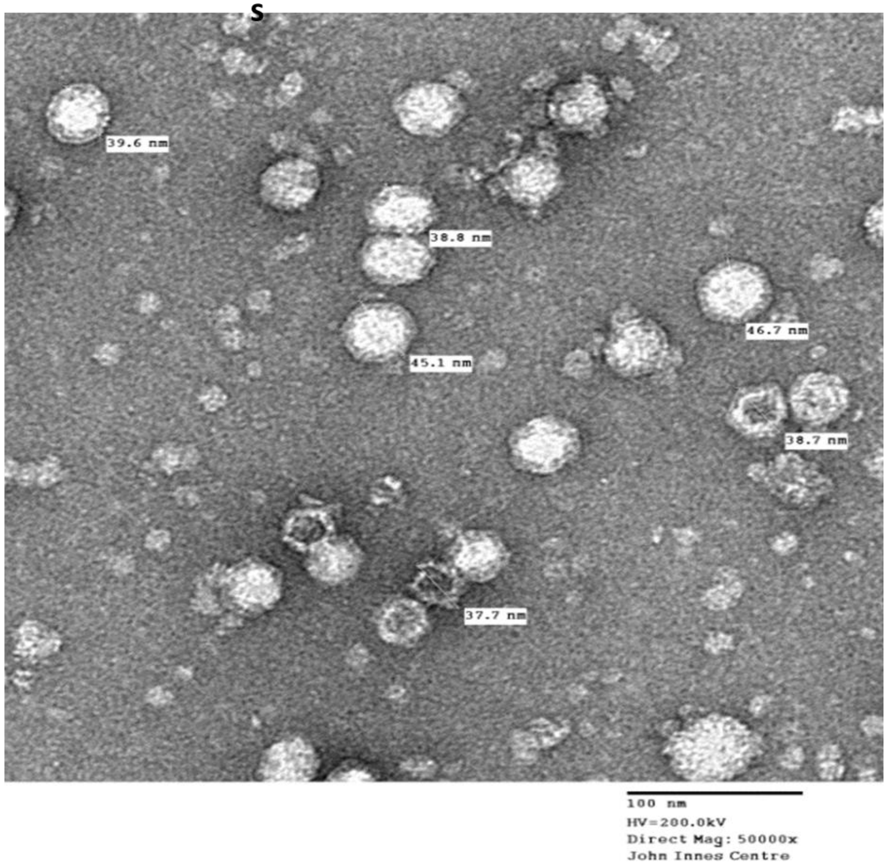

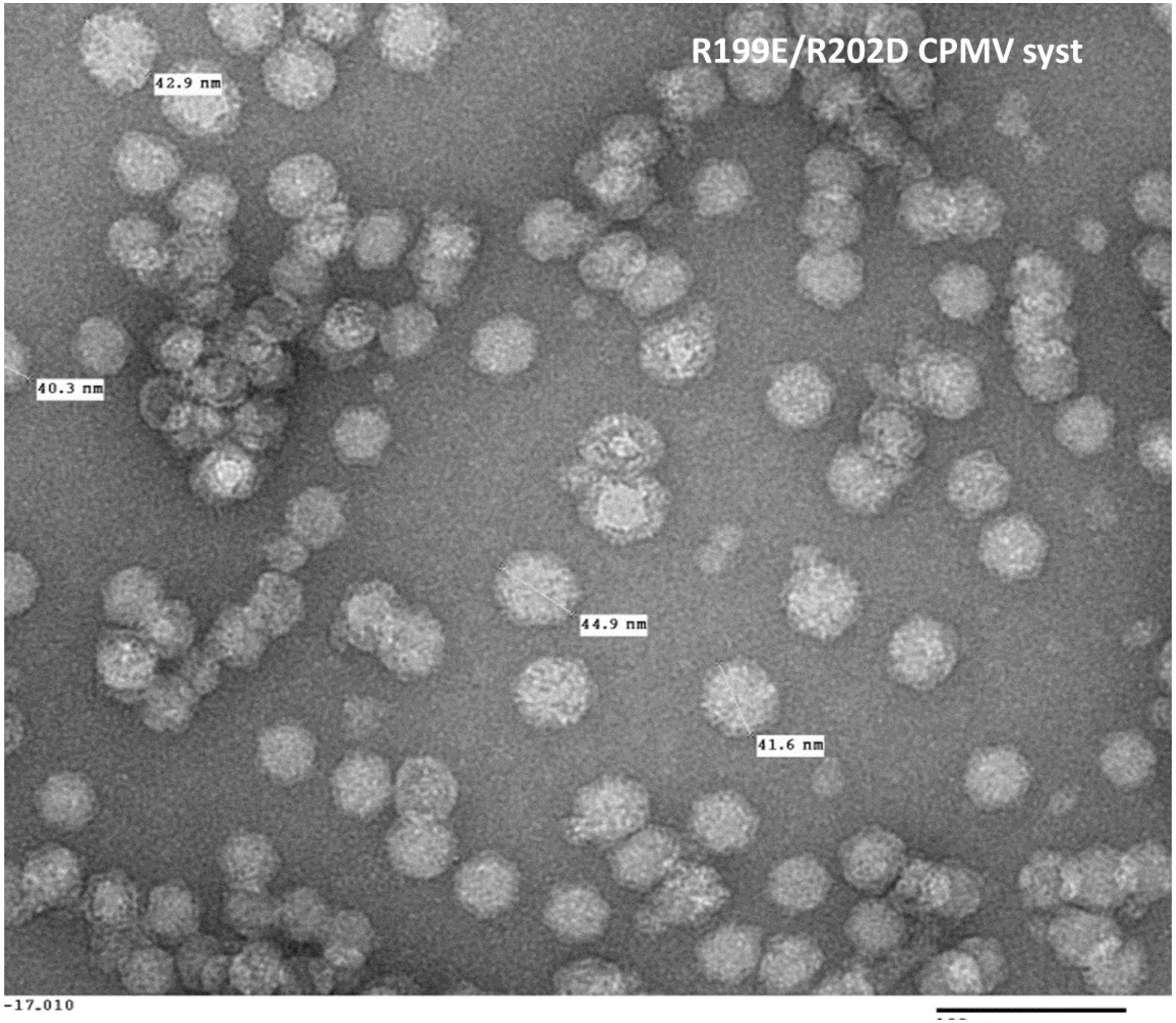

Supplementary Figure 1: Enlarged micrographs on Images shown in Figs. 1 and 2 showing the measured diameters of individual particles.
